# EMDR as a treatment option for conditions other than PTSD

**DOI:** 10.1192/j.eurpsy.2023.312

**Published:** 2023-07-19

**Authors:** H. Abrebak, F. Z. Chamsi, A. Essafi, A. Taqui, S. Radi, A. El ammouri

**Affiliations:** Psychiatric department, faculty of medicine of tangier, Tangier, Morocco

## Abstract

**Introduction:**

Eye movement desensitization and reprocessing (EMDR) is a psychotherapeutic approach that has been shown to be effective in the treatment of post-traumatic stress disorder (PTSD). The technique is known to facilitate the reprocessing of maladaptive memories thought to be at the heart of this pathology.

Strong evidence shows that traumatic events can contribute to the onset of serious mental disorders and can worsen their prognosis.

Therefore, research on EMDR therapy has increased beyond PTSD and several studies have analyzed the effect of this therapy in other mental health conditions such as psychosis, bipolar disorder, depression, anxiety disorders, substance use disorders and chronic pain.

**Objectives:**

The objective of this systematic review is to summarize the most important results of available studies conducted in this area.

**Methods:**

We performed a systematic literature search among PubMed, ScienceDirect and Scopus. Studies included work published up to 2021

The search was performed automatically by title in each database and included the keywords “EMDR”, “Eye Movement Desensitization and Reprocessing” excluding those focusing on trauma and PTSD

**Results:**

Studies are still sparse in these comorbid conditions, but available evidence suggests that EMDR therapy improves trauma-associated symptoms and has a minor effect on primary disorders by achieving partial symptomatic improvement. A positive effect has been reported in many pathological situations, including addictions, somatoform disorders, sexual dysfunctions, eating disorders, adult personality disorders, mood disorders, severe stress reaction, anxiety disorders, pain, neurodegenerative disorders, mental disorders of childhood and adolescence and sleep.

**Conclusions:**

Despite a generally positive view of EMDR as an alternative treatment option, more methodologically rigorous studies are needed.

**Disclosure of Interest:**

None Declared

